# Interpretable machine-learning-based prediction of postpartum haemorrhage in normal vaginal births in Shanghai, China

**DOI:** 10.3389/fmed.2025.1670987

**Published:** 2025-10-15

**Authors:** Xiao Yao, Yirong Bao, Na Wu, Shanshan Shan, Yiting Xu, Keying Huo, Rong Huang, Hao Ying

**Affiliations:** Shanghai Key Laboratory of Maternal Fetal Medicine, Shanghai Institute of Maternal-Fetal Medicine and Gynecologic Oncology, Shanghai First Maternity and Infant Hospital, School of Medicine, Tongji University, Shanghai, China

**Keywords:** postpartum haemorrhage, vaginal delivery, models, machine learning, factor

## Abstract

**Background:**

Postpartum haemorrhage is the most common complication associated with vaginal birth and a principal cause of maternal mortality. While clinical guidelines suggest that the majority of postpartum haemorrhage cases can be averted through precise prediction and scientific management that utilise assessment tools, existing tools for predicting postpartum haemorrhage in vaginal births have demonstrated inadequacies.

**Aim:**

To develop a predictive model for postpartum haemorrhage in vaginal births based on machine-learning algorithms.

**Methods:**

We selected pregnant women who gave birth vaginally at a tertiary-level obstetrics and gynaecology hospital in Shanghai, China, from July 2023 to August 2024. Multidimensional data were collected on demographic factors of pregnant women and midwives, along with their antenatal factors (e.g., previous medical history, current medical history, laboratory indicators, and psychosocial factors) and intrapartum factors (e.g., induction techniques; the first, second, and third stages of labour; and other factors). Five predictive models were constructed using machine-learning algorithms, and these models were subsequently validated and evaluated for performance. We applied the SHapley Additive exPlanations tool to conduct an interpretative analysis of the optimal model.

**Findings:**

A total of 1,225 women who underwent vaginal births were included in our final analysis, and following univariate analysis and least absolute shrinkage and selection operator regression, 13 predictive variables were incorporated into the model. The eXtreme Gradient Boosting model exhibited the most superior performance. A midwife’s years of service, degree of a woman’s fear of childbirth, parity, duration of the second stage of labour, episiotomy, and companionship during labour and childbirth were identified as significant predictive factors. Moreover, the midwife’s years of service and their companionship during childbirth had a moderating effect, which could effectively reduce the impact of childbirth fear and prolonged labour on the risk of postpartum haemorrhage.

**Conclusion:**

The postpartum haemorrhage prediction model for vaginal births developed in this study will furnish clinical midwives with a scientific and objective tool for assessing the risk of postpartum haemorrhage, thereby supporting timely risk stratification and management in the immediate postpartum period.

## 1 Background

Postpartum haemorrhage (PPH) exerts a severe impact on women’s reproductive and mental health, leads to increased medical expenditures, and is a leading cause of maternal mortality—accounting for approximately 27% of global maternal deaths (1). Data from the World Health Organization (WHO) indicate that over 70% of all births globally are vaginal births (2), making this the predominant mode of birth. However, PPH can follow vaginal birth and is defined as vaginal blood loss of ≥ 500 mL within 24 h after the birth of the foetus, or blood loss accompanied by signs or symptoms of hypovolaemia (3, 4). PPH is the most common complication associated with vaginal birth, with an incidence of 3.2% in the United States (5), 8.0% in Australia (6), and 16% in Nepal (7). Since 1978, China’s fertility policy has undergone several adjustments, from the “one-child policy” to the “universal two-child policy” and then to the “universal three-child policy.” We have observed an increase in the number of pregnancies among women of advanced maternal age, along with a rise in the incidence of pregnancy complications and comorbidities, with the incidence of PPH also rising annually. Data from the National Maternal Quality Control Centre for Obstetrics in China show that from 2016 to 2020, the incidence of PPH in tertiary hospitals increased from 3.8 to 10.4%, and that it remains the foremost complication within the spectrum of vaginal birth complications (8). Both the International Federation of Gynaecology and Obstetrics (FIGO) guidelines on the management of PPH in 2022 (3) and the Chinese Medical Association’s guidelines on the prevention and treatment of PPH in 2023 (4) signify that by fully identifying the factors that influence PPH and by applying risk-assessment tools for precise prediction and scientific management, PPH can be effectively prevented and the safety of childbirth for pregnant women can be ensured.

Commonly used PPH assessment tools include the California Maternal Quality Care Collaborative (CMQCC), the Association of Women’s Health, Obstetric and Neonatal Nurses (AWHONN), and the PPH prediction score sheet developed by the Chinese PPH Prevention and Treatment Collaboration Group (CPPTCG) (9–11). A study that encompassed 7,015 cases of women undergoing caesarean section showed that the areas under the receiver operating characteristic Area Under the Curves (AUCs) for the CMQCC and AWHONN assessment sheets were 0.70 and 0.69, respectively (12). Another study that comprised 11,679 cases of vaginal birth indicated that the AUC values for the CMQCC and AWHONN assessment sheets were 0.50 and 0.61, respectively (13)—lower than those for the caesarean section population—revealing that the predictive performance for the vaginal birth population was unsatisfactory. With the changes in fertility policies and concepts, disease risk factors, and treatment measures, and the clinical application of new labour stages, the accuracy of the PPH-prediction score sheet developed by the CPPTCG has gradually declined (14). While scholars have subsequently developed predictive models for PPH in vaginal births, the variables included in the extant models focus chiefly on obstetric- and delivery-related factors of pregnant women, with Logistic Regression (LR) models being the most common, and the optimal model reflects an AUC value of 0.873 (15–19). Investigators have demonstrated that the psychosocial factors of pregnant women (20, 21), the midwife’s years of service, companionship during labour, and delayed umbilical cord cutting (22) are the primary elements influencing PPH in vaginal births. It is thus evident that the influencing factors included in existing predictive models are deficient, and that the accuracy of the models needs to be further improved. LR is straightforward to implement and interpret, and it remains robust even with small samples. However, its linear assumptions and reliance on manually crafted features hinder the full exploitation of complex interactions between variables (23). Machine-learning algorithms possess powerful data-processing capabilities and can mine data and deeply analyse the potential relationships between variables, and can thereby be adopted to accurately predict the development and outcomes of clinical diseases (24).

In light of the above, based on a review of the literature, we herein incorporated multidimensional data that included demographic factors of pregnant women and midwives, antenatal factors (e.g., previous medical history, present medical history, laboratory indicators, and psychosocial factors), and intrapartum factors (e.g., induction techniques; the first, second, and third stages of labour; and other factors). Using machine-learning algorithms, we generated five predictive models for PPH in vaginal births: LR, Support Vector Machine (SVM), Random Forest (RF), Artificial Neural Network (ANN), and eXtreme Gradient Boosting (XGBoost). The SHapley Additive exPlanations (SHAP) tool was also exploited to elucidate the contribution of the feature variables in the optimal model and to thoroughly investigate the interplay of the influencing factors. We posit that our results will assist clinical medical staff in identifying high-risk groups for PPH in vaginal births, shift the prevention and control of PPH forward, strengthen management, and ensure birth safety.

## 2 Methods

### 2.1 Study design

This was a prospectively recruited observational cohort, with continuous follow-up until 24 h postpartum. While enrolment and baseline data were prospective, some predictors were only available intrapartum or postpartum; thus the model incorporates both prospective and retrospective elements. The study adhered to the TRIPOD+AI guidelines (25) (please refer to TRIPOD+AI checklist). Our study was conducted in strict accordance with ethical standards (having received approval from the hospital’s Ethical Review Committee [reference number KS223257] and with the principles of informed consent, voluntariness, and non-maleficence.

### 2.2 Study setting

The study was conducted at a tertiary-level obstetrics and gynaecology hospital in Shanghai that annually admits approximately 10,000 women experiencing vaginal birth. The labour suite is staffed with two resident obstetricians who manage critical care for pregnant women, handle emergencies, and perform instrumental vaginal births. Furthermore, 49 midwives are responsible for monitoring and managing the labour process, providing analgesic care during childbirth, accompanying women in labour, supporting free body positions during labour and birth, conducting normal vaginal births, suturing wounds, providing early neonatal care, assisting with balloon placement and artificial rupture of membranes for induction, administering oxytocin for labour induction, and participating in emergency interventions for various PPH.

### 2.3 Participants

We employed convenience sampling to select women admitted to the hospital for labour but had not yet gone into labour between July 2023 and August 2024. The inclusion criteria were as follows: ① chronological age of 20 years or above, ② carrying a single foetus, ③ harbouring a gestational age of 28 weeks or more, ④ intending to undergo a vaginal birth, and ⑤ possessing good communication skills and the ability to complete questionnaires independently and sign an informed consent form. The exclusion criteria were as follows: ① having disorders of consciousness or psychiatric illnesses, ② a planned instrumental vaginal birth or caesarean section, ③ women who requested to withdraw from the study during its implementation.

The sample size required for this study was calculated using the formula: N = (Z^2^ * p * [1 − p]) / E^2^ (26). Assuming a 95% confidence interval, we used the following values: Z (Z-score for a 95% confidence interval) = 1.96. A review of prior literature revealed that the incidence of PPH following vaginal delivery in China ranges from 3.8 to 10.4% (8). Therefore, p (proportion) = 0.10 and E (margin of error) = 2% (26, 27). Based on these parameters, the calculated sample size (*N*) was 875, and considering a 10% attrition rate, our final sample size was determined to be 963 cases.

### 2.4 Measurement indicators and tools

#### 2.4.1 Predictors

We systematically reviewed relevant literature and, in conjunction with clinical practice and research requirements, developed the “PPH Data Collection Form for Vaginal Births.” Its contents include 55 influencing factors related to demographic factors of pregnant women and midwives, as well as their antenatal factors (including previous medical history, present medical history, laboratory indicators, and psychosocial factors) and intrapartum factors (including induction techniques; the first, second, and third stages of labour; and other factors). Anxiety scores were assessed using the Generalized Anxiety Disorder Scale-7 (GAD-7), depression scores using the Edinburgh Postnatal Depression Scale (EPDS), and childbirth fear scores using the Childbirth Attitude Questionnaire (CAQ).

The Generalized Anxiety Disorder Scale-7 (GAD-7) was developed by Spitzer et al. (28) in 2006, based on the diagnostic criteria for the Generalized Anxiety Disorder in the Diagnostic and Statistical Manual of Mental Disorders, and the scale comprises 7 items, with each scored from 0 to 3 for a total of 21 points. Scores ranging from 0 to 4 indicated no anxiety, 5 to 9 indicated mild anxiety, 10 to 14 indicated moderate anxiety, and 15 to 21 indicated severe anxiety. The scale showed a Cronbach’s *α* coefficient of 0.92 and a test–retest reliability of 0.8, thus demonstrating good applicability for women in both early and late pregnancy (29).

The Edinburgh Postnatal Depression Scale (EPDS) was developed by Cox et al. (30), and consists of 10 items with each scored from 0 to 3 for a total of 30 points, with a diagnostic cutoff of 13 points indicating a depressive state. The scale has a Cronbach’s *α* coefficient of 0.87, a split-half reliability of 0.88, and is widely used for prenatal depression screening in late pregnancy (31).

The Childbirth Attitude Questionnaire (CAQ) is based on a clinical study of childbirth fear in the third trimester of pregnancy from 1982, and the scale was adapted by Tanglakmankhong et al. (32). Wei et al. (33) translated and sinicized the CAQ to form the Chinese version of the Childbirth Fear Inventory that consists of 16 items, with each scored from 1 to 4, with a minimum score of 16 and a maximum of 64. Scores from 16 to 27 indicated no fear of childbirth, 28 to 39 indicated mild fear, 40 to 51 indicated moderate fear, and 52 to 64 indicated severe fear. The scale reflected a Cronbach’s *α* coefficient of 0.91.

#### 2.4.2 Outcome

The outcome was defined as whether PPH would occur in women undergoing vaginal delivery. According to the FIGO guidelines and the Chinese Medical Association’s guidelines, PPH was defined as vaginal blood loss of ≥500 mL within 24 h after the delivery of the fetus (3, 4). Blood loss was assessed using a combination of the volumetric and gravimetric methods.

### 2.5 Data-collection methods

Participants were carefully selected based on stringent inclusion and exclusion criteria, and each participant provided informed consent prior to data collection. The research team consisted of the principal investigator, five midwives, and two students. All team members underwent standardized training to ensure consistency in data collection. The data collection process was divided into three main components: ① Questionnaire Survey: When a patient was admitted to the hospital for expectant management before the onset of labour, a paper-based questionnaire was distributed by the researcher in the obstetrics ward. This questionnaire aimed to gather information on the demographic and psychosocial factors of the pregnant women. Completed questionnaires were immediately collected and reviewed for completeness and accuracy. Any missing or incorrect information was promptly supplemented and corrected with the assistance of the participating women. ② Medical history data collection: Once the participants were admitted to the delivery room, the research team members reviewed their medical histories to collect data on antenatal factors. This included previous medical history, current medical history and laboratory indicators. The laboratory indicators were compiled from the most recent tests conducted prior to labour. ③ Delivery information collection: The research team members continuously monitored the participants throughout the labour process to dynamically collect data on intrapartum factors. This included information on induction techniques, the first, second, and third stages of labour; other intrapartum factors, and blood loss data.

### 2.6 Algorithm introduction

We employed a variety of supervised learning algorithms suitable for binary classification to construct a predictive model for PPH following vaginal delivery. The algorithms utilised include LR, RF, SVM, XGBoost, and ANN. LR is a simple and classic algorithm. RF is a supervised learning algorithm based on decision trees, capable of performing effective classification and regression analysis, thereby providing more accurate and stable predictions. Moreover, SVM can achieve highly accurate predictions using flexible nonlinear kernels. XGBoost is an enhanced gradient boosting algorithm, which is more efficient, improves precision, and has gained significant attention and application value. Finally, ANN, mimicking the information processing characteristics of biological neural networks, is widely applied in medical fields such as disease diagnosis, prediction, and classification (23, 24, 34).

### 2.7 Statistical methods

Statistical analysis: Data analysis was performed with SPSS20.0, and Python3.13 was used for data cleaning, statistical analysis and graphing. Parametric statistical methods were applied to data that conformed to a normal distribution and are presented as^−^x ± s, with group differences assessed via independent—with effect size estimated using Cohen’s d. For data that did not follow a normal distribution, values are presented as M (Q1, Q3), and group comparisons were conducted using the Mann–Whitney U test—with effect size estimated using *r*. Categorical data were depicted as percentages (%), with group differences evaluated using Chi-squared tests, and effect size was estimated using *φ*. A two-sided test was used, with *p* < 0.05 indicating statistically significant differences.

Variable selection: To effectively handle high-dimensional data and mitigate the risk of overfitting (35), we conducted univariate analysis and employed Least Absolute Shrinkage and Selection Operator (LASSO) regression for variable selection in the training dataset. Variables with statistically significant differences between the PPH group and the non-PPH group were included in the LASSO regression for variable selection, with the onset of PPH as the outcome variable.

Model construction and performance evaluation: We evaluated five algorithms (LR, RF, XGBoost, SVM, ANN). All patients enrolled in this study were divided into a training dataset and testing dataset in an 80:20 ratio using stratified sampling. After hyperparameters were tuned via fivefold cross-validation on the training dataset, a single final model was retrained on the entire training dataset with the optimal parameters, and this model was then applied to the independent test dataset to generate the reported results. The predictive performance of the models was evaluated using AUC, accuracy, recall, precision, F1 score, and area under the precision–recall curve (AUPRC).

Model interpretability: The SHAP tool was ultimately exploited to conduct an interpretative analysis of the decision-making role of feature variables in the model. Specifically, this study utilised SHAP summary plots and bee swarm plots for global interpretation, while SHAP interaction and dependence plots were used to illustrate feature correlations. Moreover, we performed an interaction effect analysis with the traditional LR model.

## 3 Results

We enrolled 1,304 participants prior to the commencement of labour upon admission to the maternity ward. Of these, 53 women eventually underwent caesarean sections, and 26 required instrumental assistance during the birthing process; this led to a final study population of 1,225 cases. The dataset was divided into a training dataset and a test dataset at an 80:20 ratio using stratified sampling, resulting in 980 cases for the former and 245 cases for the latter. Within the training dataset, there were 248 instances of PPH, while within the test dataset, there were 62 instances. The stratified sampling diagram of the research subjects is shown in Supplementary material 1.

### 3.1 Variable selection

Comparisons were made in the training dataset between women who experienced PPH and those who did not, and 25 variables exhibited statistically significant differences (*p* < 0.05): gestation age, gravidity, parity, gestational weight increment, degree of childbirth fear, advanced maternal age, diagnosis of liver impairment, prenatal diagnosis of macrosomia, amniotic fluid contamination, antepartum fever, Cook’s Cervical Ripening Balloon, duration of the first and second stages of labour, episiotomy, mode of placental delivery, neonatal birth weight, placenta retention, blood type, white blood cell count, activated partial thromboplastin time (APTT), midwife’s years of service, companionship during labour and childbirth, epidural analgesia, maternal mobility and position, and delayed cord clamping. The remaining variables did not differ (*p* > 0.05, as detailed in Supplementary material 2).

**Figure 1:**
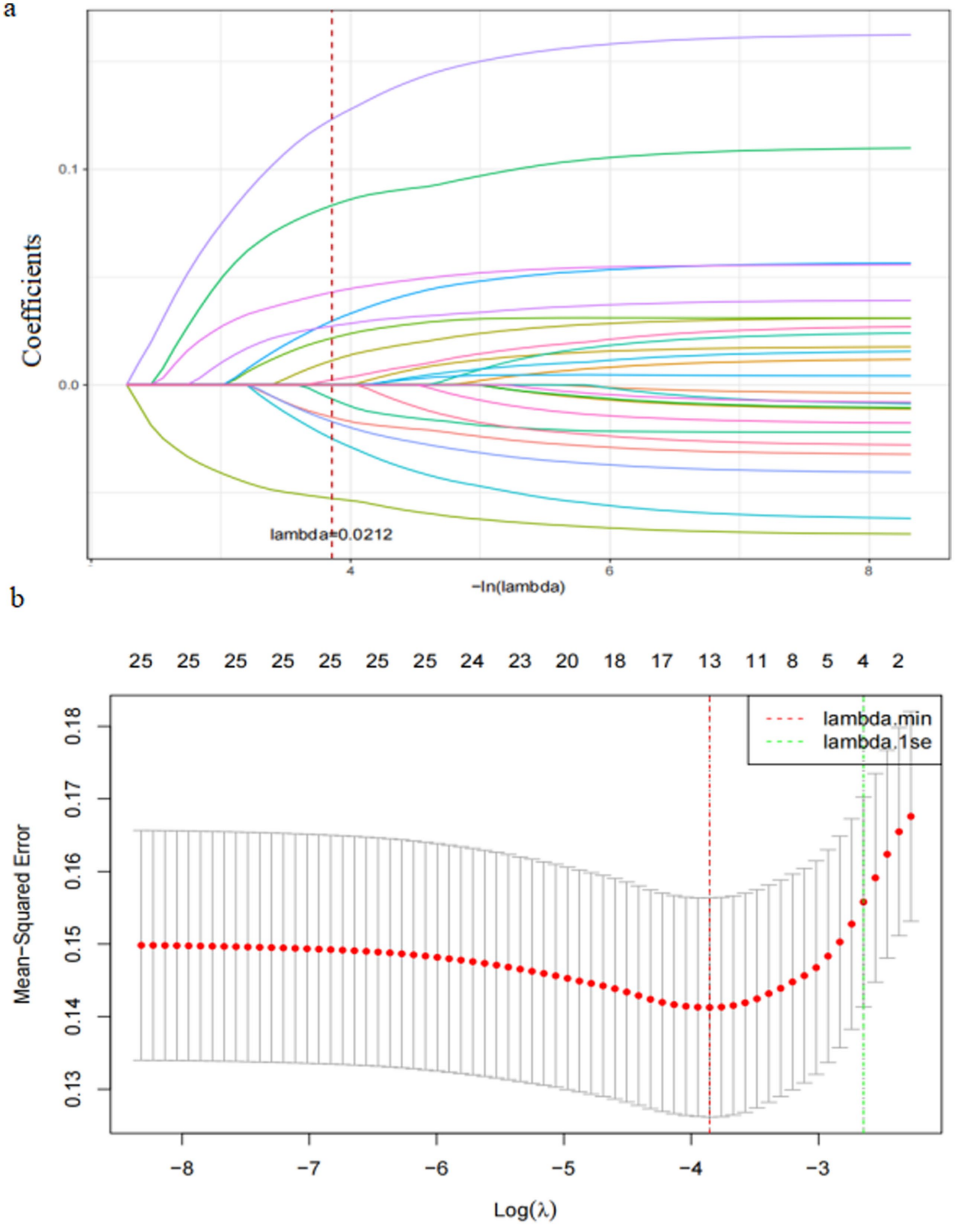
The process of variable selection based on LASSO regression. Panel **(a)** is the changes in the coefficients of all independent variables as a varies with log (*λ*); panel **(b)** is the process of variable selection.

The 25 variables that demonstrated statistical significance between the PPH and non-PPH groups within the training dataset underwent analysis through LASSO regression; and the variable selection process facilitated by LASSO regression is visually represented in Figure 1. Figure 1A depicts the inverse relationship between the penalty parameter (*λ*) and the magnitude of coefficient shrinkage for the model’s independent variables, and culminated in the progressive elimination of variables. Figure 1B delineates the graphical relationship between the count of retained independent variables and the logarithm of the penalty parameter (*λ*) as marked by two dashed lines denoting λ (min) and λ (1 se). The λ (min) threshold identified the optimal model coefficients at the lowest mean squared error, and encompassed 13 independent variables; whereas *λ* (1 se) denoted the optimal model coefficients that fell within one standard error of the minimal mean squared error and accounted for four variables. The 13 parameters deemed most influential as determined by λ (min) were integrated into the predictive model: gravidity, parity, companionship during labour and childbirth, episiotomy, mode of placental delivery, duration of the second stage of labour, midwife’s years of service, diagnosis of liver impairment, prenatal diagnosis of macrosomia, antepartum fever, blood type, delayed cord clamping, and the degree of childbirth fear. These 13 variables showed no statistically significant differences between the training and test datasets, as detailed in Supplementary material 3.

### 3.2 Model construction

The 13 variables identified through LASSO regression were incorporated into the predictive models. Within the training dataset, five predictive models were developed using grid search with fivefold cross-validation to optimise the hyperparameters: LR, RF, XGBoost, SVM, and ANN. The specific hyperparameters for each model are detailed in Supplementary material 4. All models demonstrated an AUC value greater than 0.8, with XGBoost achieving the highest at 0.834; and accuracy rates exceeded 0.8 for all models, peaking at 0.816 for RF. Precision rates were all greater than 0.7, with XGBoost leading at 0.764. F1 scores, which balance precision and recall, were all greater than 0.5, with RF attaining the highest value of 0.549; and recall rates were all greater than 0.4, with ANN showing the highest at 0.454. It was evident that all five models held considerable predictive value for PPH following vaginal birth, with RF and XGBoost models showing particularly strong predictive performance (detailed results are presented in Supplementary material 5).

### 3.3 Model performance evaluation

**Figure 2:**
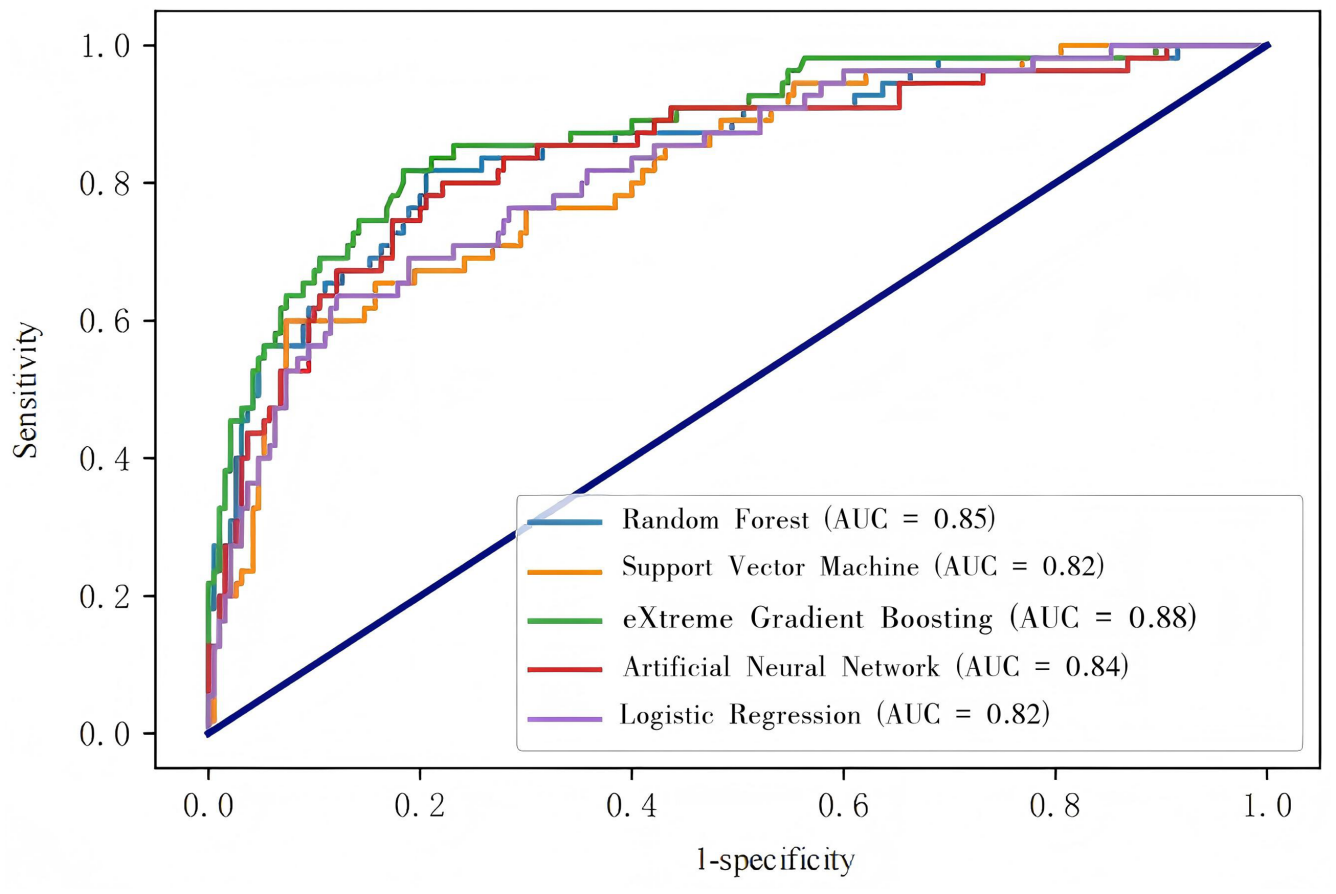
The AUCs of the five machine- learning models in the test set.

**Figure 3:**
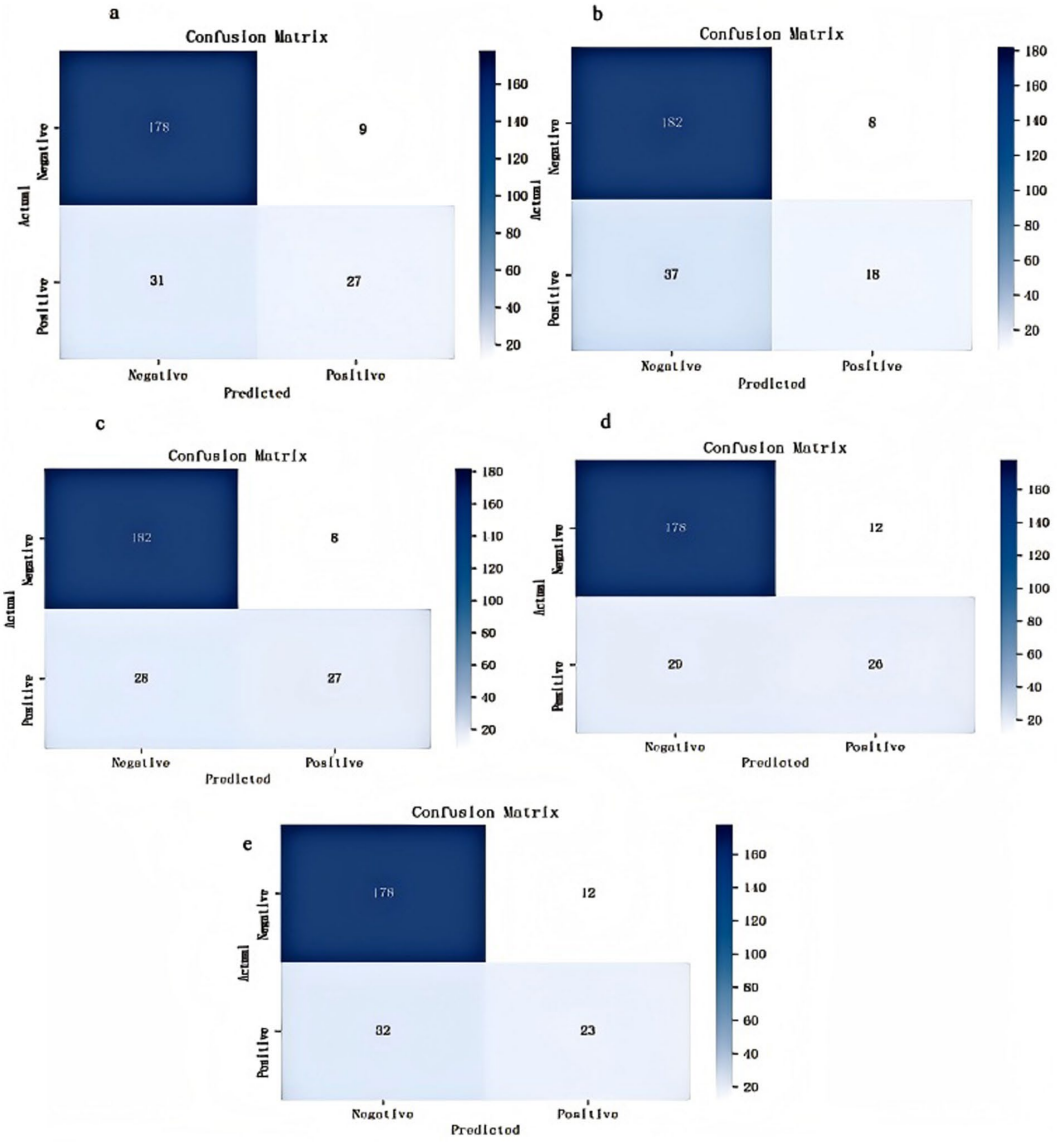
The confusion matrices of the five machine-learning models in the test set. Panel **(a)** random forest, panel **(b)** support vector machine, panel **(c)** eXtreme gradient boosting, panel **(d)** artificial neural network, panel **(e)** logistic regression.

**Figure 4:**
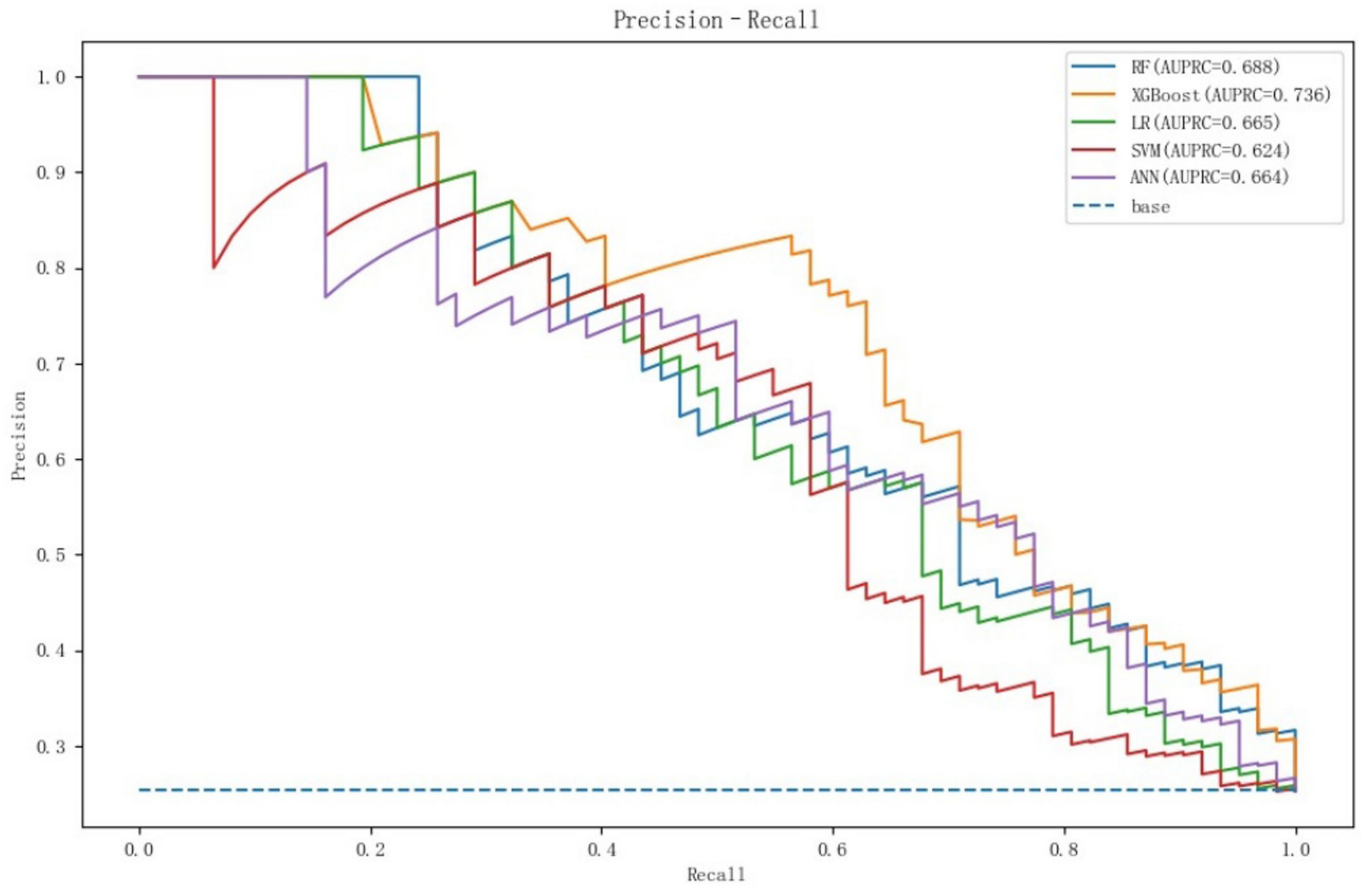
The AUPRC for each model are in the test set.

Our models were evaluated on the test dataset, and the outcomes revealed that all 5 models possessed an AUC above 0.8, with XGBoost achieving the highest score of 0.877 (the AUCs for each model are depicted in Figure 2). The confusion matrices indicated high values for both true positives (TP) and true negatives (TN), with XGBoost models ranking highest (TP = 27, TN = 182; Figure 3). The AUPRC above 0.6, with XGBoost achieving the highest score of 0.736 (the AUPRC for each model is depicted in Figure 4). All models achieved an accuracy greater than 0.80, F1 score above 0.40, recall above 0.30 and precision above 0.40, with XGBoost attaining the highest values across these metrics (a summary of the performance metrics for each model is detailed in Table 1). Considering all evaluation metrics, the XGBoost model emerged as the optimal predictor for PPH following vaginal birth.

**Table 1:** The performance evaluation metrics of the five machine-learning algorithms.

Model	AUC	Accuracy	Precision	F1 score	Recall
RF	0.852	0.836	0.750	0.574	0.465
SVM	0.815	0.816	0.692	0.444	0.327
XGBoost	0.877	0.853	0.771	0.600	0.491
ANN	0.840	0.833	0.684	0.559	0.473
LR	0.820	0.820	0.657	0.511	0.418

RF, Random Forest; SVM, Support Vector Machine; XGBoost, eXtreme Gradient Boosting; ANN, Artificial Neural Network; LR, Logistic Regression.

### 3.4 Interpretability analysis of the model

**Figure 5:**
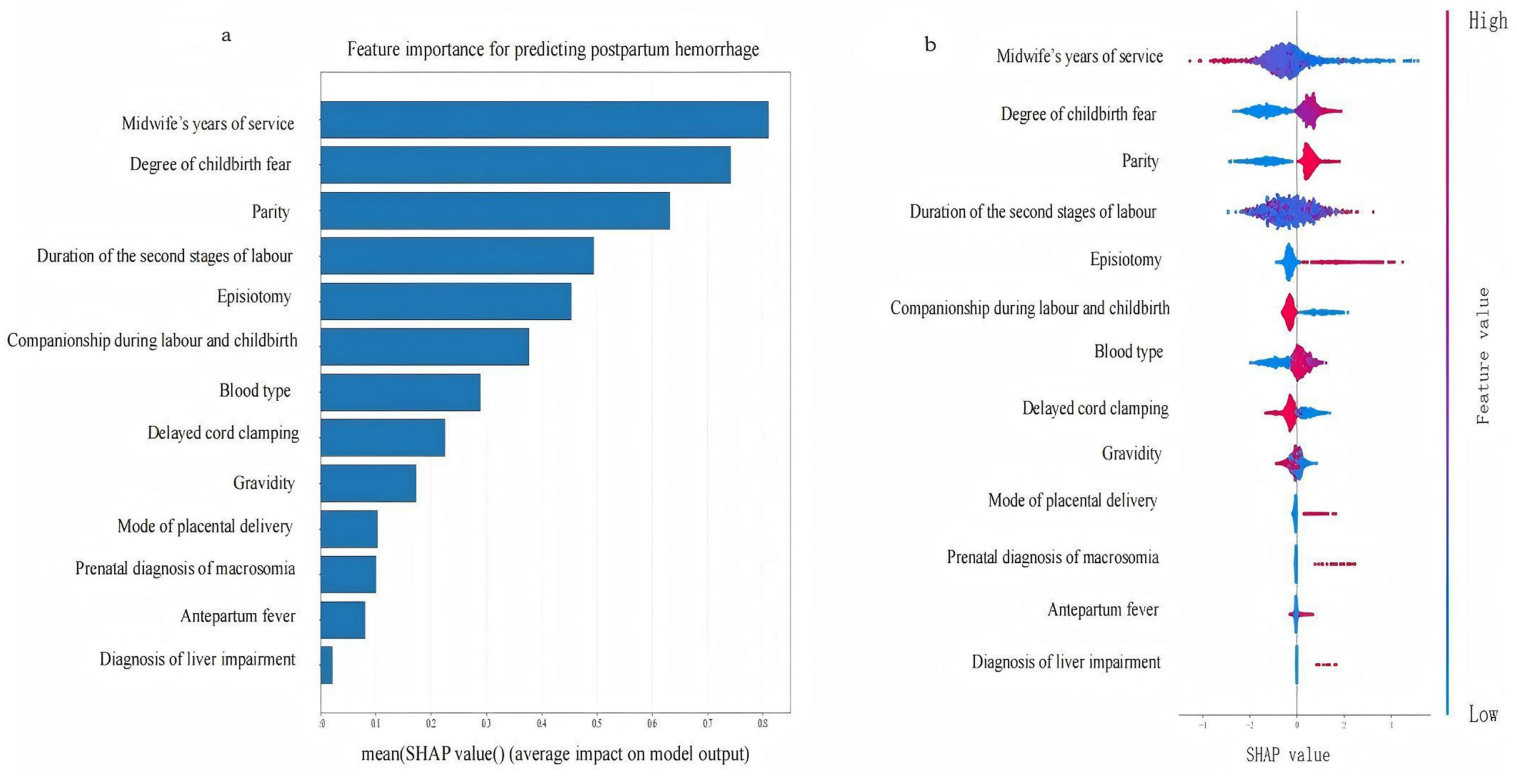
The SHAP for the feature variables of the XGBoost. Panel **(a)** is the feature importance plot, Panel **(b)** is the SHAP bee swarm plot.

**Figure 6:**
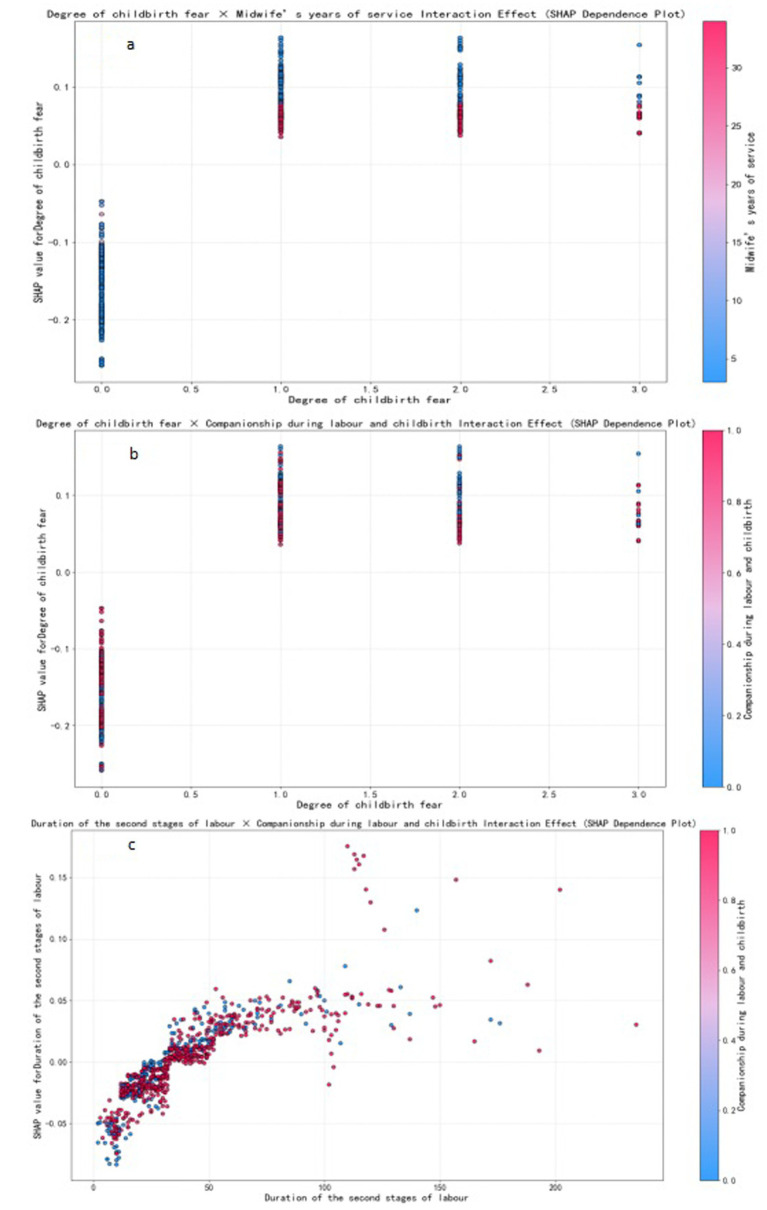
SHAP dependence plots. Individual data points represent samples, color-coded to indicate the magnitude of feature values- red for high and blue for low.

We selected for analysis the optimal model for PPH following vaginal birth, i.e., XGBoost; and the SHAP tool was employed to rank the importance of the 13 feature variables included in the model and to visualise the extent of each feature’s impact on the model’s predictions. Our results indicated the following order of importance from high to low for the 13 feature variables: midwife’s years of service, degree of childbirth fear, parity, duration of the second stage of labour, episiotomy, companionship during labour and childbirth, blood type, delayed cord clamping, gravidity, method of placental delivery, prenatal diagnosis of macrosomia, antepartum fever, and diagnosis of liver impairment. Lower values for the midwife’s years of service, companionship during labour and childbirth, delayed cord clamping, and gravidity were associated with a higher likelihood of the model predicting PPH; conversely, higher values for the remaining feature variables indicated a greater likelihood of the model predicting PPH (the SHAP for the XGBoost model’s features is shown in Figure 5). Further analysis revealed that the midwife’s years of service exhibited interaction effects with the degree of childbirth fear. Companionship during labour and childbirth showed interaction effects with the degree of childbirth fear and the duration of the second stage of labour (SHAP dependence plots is shown in Figure 6). Moreover, the interaction effect was determined using the traditional LR model (the detailed results are presented in Supplementary material 6).

## 4 Discussion

Most published PPH prediction models have focused on cesarean deliveries, while only a minority have addressed vaginal deliveries, and the majority of these have been based on logistic regression. We herein focused on the prediction of PPH following vaginal childbirth, and constructed predictive models based on machine-learning algorithms by analysing pertinent data. Our investigation encompassed a multifaceted data collection and employed univariate analysis and LASSO regression to select feature variables. Five predictive models were generated using machine-learning algorithms and then compared. The results indicated that the RF, SVM, XGBoost, ANN, and LR models were all suitable for predicting the risk of PPH following vaginal childbirth, with favourable predictive performance. However, the XGBoost model demonstrated the best performance, with an AUC of 0.877, AUPRC of 0.736, accuracy of 0.853, precision of 0.771, F1 score of 0.600, and recall rate of 0.491. The confusion matrix revealed that among the 245 cases in the test set, the XGBoost model correctly predicted 209 cases. The model’s predictive performance was higher than that of the existing PPH prediction models for vaginal delivery (15–19). Liu et al.’s (16) construction of RF, K Nearest Neighbour (KNN), and an integrated model with Light Gradient Boosting Machine (LGB) and LR outperformed traditional LR models—with the LGB + LR model achieving the best predictive performance for PPH, eliciting an AUC value of 0.803. Previous studies that relied solely on clinical variables to build LR models reported AUCs of 0.708, 0.660, 0.860 and 0.873 (15, 17–19) (details are provided in the Supplementary material 7). This discrepancy likely stems from two factors: first, the previous studies were restricted to a limited set of predictors, with some models using only basic clinical data. Second, the algorithms for constructing models in this regard still need improvement. Therefore, we used machine-learning techniques to expand the feature space beyond clinical variables by incorporating maternal psychosocial factors of pregnant women, demographic factors of the midwives, and midwife-related factors during labour to develop more robust prediction models.

Multivariate studies have consistently identified parity, the duration of the second stage of labour, episiotomy, gravidity, the mode of placental delivery, prenatal diagnosis of macrosomia, and antepartum fever as determinants of PPH after vaginal delivery (17, 36–38). These conventional clinical variables were likewise confirmed as significant predictors in the present cohort. In contrast to earlier work, our model further incorporates and demonstrates the prognostic relevance of midwife’s years of service, companionship during labour and childbirth, delayed cord clamping, degree of childbirth fear, blood type, and diagnosis of liver impairment (description of the variables in the Supplementary material 8). Among these, the midwife’s years of service, companionship during labour and childbirth, and delayed cord clamping were protective factors, while the remaining variables were identified as risk factors. Moreover, the midwife’s years of service exhibited interaction effects with the degree of childbirth fear. Companionship during labour and childbirth also showed interaction effects with the degree of childbirth fear and the duration of the second stage of labour. These interactions played a crucial role in reducing the incidence of PPH following vaginal delivery.

Of these, the midwife’s years of service was considered the most important influencing factor: the longer the midwife’s years of service, the lower the incidence of PPH. A group from Kenya previously explored the midwife-related factors that affected the management of PPH, and their results showed that midwives with 5 years of service exerted a significantly positive impact on the management of PPH (39). Additionally, this study also found that the midwife’s years of service had a moderating effect on the degree of childbirth fear. When pregnant women experience childbirth fear, midwives with more extensive experience can significantly reduce the impact of childbirth fear on the risk of PPH. Previous studies have shown that more years of service among midwives can mitigate the degree of childbirth fear (40), but no study has yet examined how midwife’s years of service, childbirth fear, and PPH are linked or the precise mechanisms that underpin these associations. A longer midwife tenure is associated with more sophisticated theoretical knowledge, refined technical skills, and heightened professional competence (41). One plausible explanation is that seasoned midwives foster greater feelings of safety and trust in parturients, thereby attenuating fear and, in turn, reducing PPH risk. However, rigorous investigations are needed to test this pathway. This study also found that labour companionship could reduce the incidence of PPH, which is consistent with previous findings (42). In this study, companionship during labour was found to moderate the effects of childbirth fear and the duration of the second stage of labour, thus effectively reducing the impact of these factors on the risk of PPH. Companionship during labour often takes the form of one-to-one midwifery care, which provides continuous physical and emotional support to women in labour. This support helps alleviate fear and anxiety during childbirth, thus enabling women to better cope with pain and stress, thereby reducing the degree of childbirth fear (42). During the second stage of labour, women experience extreme pain and fear, but also fascination. In this stage, being involved and receiving information and guidance are considered crucial (43). Companionship during labour during the second stage of labour can help reduce childbirth fear by providing women with timely information and guidance (e.g., childbirth techniques, perineal heat application, and position guidance), thereby reducing the incidence of PPH. Delayed cord clamping is one of the measures used to prevent PPH by “actively managing the third stage of labour” (4), and it can promote the transfer of blood from the placenta to the newborn and reduce the residual blood volume of the placenta; changes in intrauterine pressure will also stimulate uterine contractions, thereby reducing the volume of PPH (44). The International Confederation of Midwives (ICM) issued a joint statement that emphasised midwifery as playing important roles in promoting reproductive health and ensuring the safety of mothers and infants (45). It is evident that to ensure the safety and quality of vaginal birth, the key role of midwives needs to be fully leveraged. During the childbirth process, midwives should actively perform services such as companionship during labour and delayed cord clamping, assist women in alleviating contraction pain, guide negative emotions, and provide psychological support. Midwives should also continue to strengthen their professional training to comprehensively improve their theoretical level of knowledge and clinical operational skills. Experienced midwives should be encouraged to participate in the delivery process of high-risk pregnant women. This will enable them to better provide high-quality care and support for mothers and newborns and thereby effectively reduce the incidence of PPH.

The degree of childbirth fear ranked second in our optimal predictive model’s SHAP analysis, with an increase in the degree of childbirth fear before labour associated with an elevated incidence of PPH—consistent with the analysis by Zhang (46) We hypothesize that the reason for this association is the perceived relationship between the degree of childbirth fear and the activity of the central nervous system. As the degree of childbirth fear deepens, the activity of the cerebral cortex of the pregnant woman is gradually inhibited, and this leads to endocrine disorders and the reduced secretion of oxytocin that causes uterine atony, thereby increasing the incidence of PPH (47). We ascertained that women with B^+^, AB^+^, and O^+^ blood types have an increased risk of PPH relative to those with type A blood. This result contrasted, however, with previous research data by Burd et al. (48) who showed that in the United States, type O blood was a risk factor. Therefore, future research endeavours are needed to further explore the association between blood type and PPH with respect to disparate ethnic backgrounds to clarify its clinically applicative value. Liver dysfunction was also a risk factor for PPH in our analysis, and some scholars have explored the relationship between liver anomalies during pregnancy and labour outcomes; with studies showing that the incidence of PPH in patients with liver dysfunction during pregnancy was increased (49). We hypothesize that the reason for this observed relationship may be related to altered coagulatory function. We acknowledge that midwives play a crucial role in the prevention and management of PPH following vaginal birth (22). Clinical midwives are advised to institute rigorous, serial surveillance of coagulation parameters and maternal blood type; implement routine psychological screening for pregnant and postpartum women; initiate early multidisciplinary interventions for high-risk individuals; and establish a streamlined green-channel protocol with the blood bank to ensure that compatible blood products are reserved in advance, thereby minimizing the risk of PPH.

This study differed significantly from previous PPH prediction models after vaginal delivery (15–19). First, the study population excluded women who had undergone instrumental vaginal delivery, and labour was managed exclusively by midwives throughout. This process identified a set of clinical, psychosocial, and care-related factors that, while previously reported, are further confirmed in our cohort, alongside additional factors such as liver impairment and blood type, which afforded both novel empirical substrates for precision PPH prevention and a pragmatic scaffold for phased, individualized obstetric care. The predictive factors has been fully integrated into the institutional clinical pathway. At labour-ward admission, gravidity, parity, blood types, prenatal diagnosis of macrosomia, hepatic impairment, and the degree of childbirth fear were assessed. Thus, parturients with identified high-risk factors can be offered targeted midwifery services, such as continuous one-to-one labour support. During labour, the duration of the second stage and the presence of antenatal fever should be reevaluated. Based on the assessed risk level, deliveries should be assigned to midwives of appropriate seniority, episiotomy is avoided, and delayed cord clamping is performed. Following delivery, the method of placental expulsion is assessed, integrating findings from every preceding stage, and the midwife determines whether to summon the physician for intervention and activate the PPH emergency team. Subsequently, a machine-learning model will be integrated into a web application built with the Dash library in Python, delivering a practical and user-friendly decision-support tool for frontline clinicians.

## 5 Limitations

This single-centre study was limited by a modest sample size owing to time and staffing constraints. Moreover, the key predictors such as duration of the second stage of labour and delayed cord clamping are only available after delivery, the present model cannot be applied antenatally or at the onset of labour. Its use is restricted to the period immediately after cord clamping. Future work will enlarge the sample, disentangle the latent relationships among factors, shift the prediction point earlier in labour, and validate the model’s generalisability through a multicentre study.

## 6 Conclusion

The XGBoost model constructed in this study demonstrated favourable performance in terms of accuracy and precision, with robustly generalisable and predictive capabilities. The model supports timely risk stratification and management in the immediate postpartum period, which may help reduce severity and improve outcomes. In addition, our model incorporates factors that differ from those reported in previous studies and clarifies the crucial role of a midwife’s years of service and patient companionship during labour in reducing the risk of PPH. It also offers a theoretical basis for subsequent research. We suggest that future work focus on conducting multicentre, large-sample longitudinal studies based on machine-learning algorithms. Such analyses would continuously optimise and improve the model, further investigating other potential protective factors, and providing more robust support for reducing the incidence of PPH and toward safeguarding maternal and neonatal health.

## Data Availability

The original contributions presented in the study are included in the article/Supplementary material, further inquiries can be directed to the corresponding authors.
