# Supplementary material for: Interpretable machine-learning-based prediction of postpartum haemorrhage in normal vaginal births in Shanghai, China
Source: Front Med (Lausanne). 2025 Oct 15;12:1670987. doi: 10.3389/fmed.2025.1670987 (PMC12570276; doi:10.3389/fmed.2025.1670987)
Supplement: Supplementary file 1 [file Data_Sheet_1.pdf]

Supplement 1 The stratified sampling diagram of the research subjects

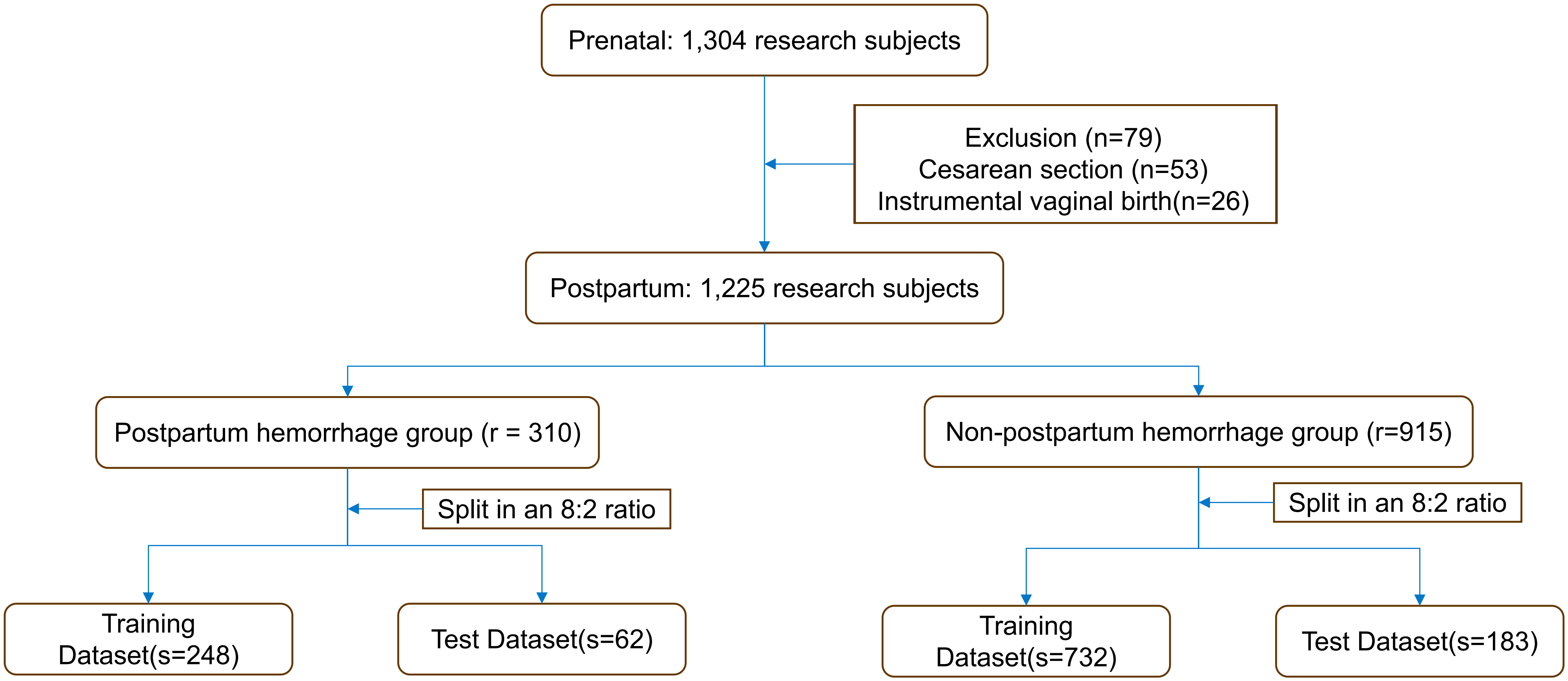

Supplement 2 Comparison of postpartum haemorrhage and non-haemorrhage data in training dataset

Characteristics	Postpartum haemorrhage group (N=248)	Non-postpartum haemorrhage group (N=732)	*t/Z/*χ^2	*Cohen'd/r/φ*	*P*
**Demographic factors of pregnant women**					
Advanced maternal age (n, %)					
Yes	24(9.68)	107(14.62)	**3.904**	**0.063**	**0.048**
No	224(90.32)	625(85.38)			
Monthly income(RMB) (n, %)					
≤5000	27（10.89）	74（10.11）	1.914	0.044	0.591
5001-10000	76（30.65）	213（29.09）			
10001-15000	75（30.24）	204（27.87）			
＞15000	70（28.22）	241（32.93）			
Education level (n, %)					
Higher education	21（8.48）	62（8.47）	1.119	0.034	0.773
Bachelor degree	164（66.13）	459（62.70）			
Master degree	63（25.39）	211（28.23）			
Occupational situation (n, %)					
Public institution	28（11.29）	88（12.02）	3.932	0.063	0.559
State-owned enterprise	35（14.11）	124（16.94）			
Foreign company	37（14.92）	107（14.62）			
Freelance work	29（11.69）	73（9.97）			
Private enterprises	110（44.35）	297（40.57）			
Unemployed	9（3.64）	43（5.91）			
Blood type (n, %)					
Type A	45(18.15)	212(28.96)	**14.775**	**0.123**	**0.002**
Type B	64(25.81)	153(20.90)			
Type AB	25(10.08)	44(6.01)			
Type O	114(45.96)	323(44.13)			
**Demographic factors of midwives**					
Age (years)	32.40±5.440	32.78±5.860	1.008	0.067	0.313
Midwife's years of service (years)	8.82±4.93	12.01±6.85	**-6.765**	**0.595**	**0.000**
Professional Title (n, %)					
Junior Professional	21(8.47)	51(6.97)	0.646	0.026	0.724
Intermediate Professional	216(87.10)	650(88.79)			
Senior Professiona	11(4，43)	31(4.24)			

Supplement 2 Comparison of postpartum haemorrhage and non-haemorrhage data in training dataset(continued)

Characteristics	Postpartum haemorrhage group (N=248)	Non-postpartum haemorrhage group (N=732)	$t/Z/\chi^2$	*Cohen'd s/r/φ*	*P*
Education level (n, %) (n, %)					
Higher education	50(20.16)	164(22.40)	0.551	0.024	0.759
Bachelor degree	195(78.63)	559(76.37)			
Master degree	3(1.21)	9(1.23)			
**Antenatal factors of previous medical history**					
Maternal smoking (n, %)					
Yes	3（1.21）	10（1.37）	0.035	0.006	0.852
No	245（98.79）	722（98.63）			
History of uterine operation (n, %)					
Yes	16（6.45）	57（7.79）	0.479	0.022	0.489
No	232（93.55）	675（92.21）			
History of PPH (n, %)					
Yes	8（3.23）	27（3.69）	0.115	0.011	0.734
No	240（96.77）	705（96.61）			
Pre-pregnancy BMI	21.39±2.915	21.66±3.012	1.354	0.091	0.175
**Antenatal factors of present medical history**					
Gravidity (n, %)					
1 time	152（61.29）	372（50.82）	**8.163**	**0.091**	**0.004**
≥2 times	96（38.71）	360（49.18）			
Parity (n, %)					
Primipara	59（23.79）	268（36.61）	**13.69**	**0.118**	**0.000**
Multipara	189（76.21）	464（63.39）			
Gestation age (n, %)					
28~36+^{6} weeks	12(4.84)	23（3.14）	**6.528**	**0.082**	**0.038**
37~42 weeks	136（54.84）	466（63.66）			
＞42 weeks	100（40.32）	243（33.20）			
Gestational weight increment (kg)	13.82±4.991	12.98±5.467	**2.132**	**0.080**	**0.033**
Assisted reproductive techniques (n, %)					
Yes	27（10.89）	88(12.02)	0.230	0.015	0.631
No	221(89.11)	644(87.98)			

Supplement 2 Comparison of postpartum haemorrhage and non-haemorrhage data in training dataset(continued)

Characteristics	Postpartum haemorrhage group (N=248)	Non-postpartum haemorrhage group (N=732)	$t/Z/\chi^2$	*Cohen'd /r/φ*	*P*
Polyhydramnios (n, %)					
Yes	1(0.40)	3(0.41)	0.000	0.000	0.989
No	247(99.60)	729(99.59)			
Myoma of uterus (n, %)					
Yes	14(5.65)	48(6.56)	0.260	0.016	0.610
No	234(94.35)	684(93.44)			
Gestational diabetes mellitus (n, %)					
Yes	24（9.68）	80（10.93）	0.306	0.018	0.580
No	224（91.32）	652（89.07）			
Diagnosis of liver impairment (n, %)					
Yes	5(2.02)	3(0.41)	**5.903**	**0.078**	**0.015**
No	243(97.98)	729(99.59)			
Intrahepatic Cholestasis of Pregnancy (n, %)					
Yes	3(1.21)	5(0.98)	0.634	0.025	0.426
No	245(98.79)	727(99.02)			
Hypertensive Disorders of Pregnancy (n, %)					
Yes	11(4.44)	40(5.46)	0.398	0.020	0.528
No	237(95.56)	692(94.54)			
Prenatal diagnosis of macrosomia (n, %)					
Yes	12(4.84)	6(0.82)	**16.595**	**0.130**	**0.000**
No	236(95.16)	726(99.18)			
Midwife clinic (n, %)					
Yes	122(49.19)	388(53.01)	1.078	0.033	0.299
No	126(50.81)	344(46.99)			
**Antenatal factors of laboratory indicators**					
Hemoglobin (g/L)	119.91±10.557	119.12±11.146	0.979	0.084	0.328
Hematocrit (%)	35.79±2.758	35.69±3.147	0.438	0.040	0.662

Supplement 2 Comparison of postpartum haemorrhage and non-haemorrhage data in training dataset(continued)

Characteristics	Postpartum haemorrhage group (N=248)	Non-postpartum haemorrhage group (N=732)	$t/Z/\chi^2$	*Cohen'd s/r/φ*	*P*
Hemameba (g/L)	10.69±4.076	10.10±3.493	**2.189**	**0.155**	**0.029**
Blood platelet (10^9/L)	185.98±49.683	191.64±49.351	-1.559	0.114	0.119
APTT rate (%)	0.92±0.068	0.91±0.055	**2.448**	**0.161**	**0.015**
Fibrinogen (g/L)	4.12±0.742	4.23±1.185	-1.475	0.111	0.141
**Antenatal factors of psychosocial factors**					
Anxiety degree (n, %)					
Normal	119(47.98)	374(51.09)	1.519	0.039	0.678
Mild	104(41.94)	297(40.57)			
Moderate	19(7.66)	50(6.83)			
Severe	6(2.42)	11(1.51)			
Depression (n, %)					
Yes	61(24.60)	155(21.17)	1.262	0.036	0.261
No	187(75.40)	577(78.83)			
Degree of childbirth fear (n, %)					
Normal	55(22.18)	331(45.22)	**41.49**	**0.206**	**0.000**
Mild	144(58.06)	306(41.80)			
Moderate	40(16.13)	79(10.79)			
Severe	9(3.63)	16(2.19)			
Postpartum haemorrhage cognition (n, %)					
Do not understand	17（6.85）	32（4.37）	4.645	0.069	0.326
Basically understand	104（41.94）	279（38.11）			
Uncertainty	77（31.05）	243（33.20）			
Clearly understand	47（18.95）	166（22.68）			
Well understand	3（1.21）	12（1.64）			
**Intrapartum factors of Induction Techniques**					
Cook's cervical ripening balloon(n, %)					
Yes	36(14.52)	157(21.44)	**5.628**	**0.076**	**0.018**
No	212(85.48)	575(78.56)			

Supplement2 Comparison of postpartum haemorrhage and non-haemorrhage data in training dataset(continued)

Characteristics	Postpartum haemorrhage group (N=248)	Non-postpartum haemorrhage group (N=732)	$t/Z/\chi^2$	*Cohen'd*/*r*/*φ*	*P*
Amniotomy (n, %)					
Yes	90(36.29)	315(43.03)	3.473	0.060	0.062
No	158(63.71)	417(56.97)			
Intravenous drip of oxytocin (n, %)					
Yes	175(70.56)	480(65.57)	2.082	0.046	0.149
No	73(29.44)	252(34.43)			
**Intrapartum factors of first stage of labour**					
Labour duration I (min)	465(327,660)	430(270,630)	**-2.511**	**0.174**	**0.012**
Amniotic fluid contamination (n, %)					
Normal	217(87.50)	686(93.72)	**9.886**	**0.100**	**0.002**
Contaminative	31(12.50)	46(6.28)			
Antepartum fever (n, %)					
Yes	53(21.37)	88(12.02)	**13.14**	**0.116**	**0.000**
No	195(78.63)	644(87.98)			
Epidural analgesia(n, %)					
Yes	233（93.96）	657（89.75）	**3.913**	**0.063**	**0.048**
No	15（6.04%）	75（10.25）			
**Intrapartum factors of second stage of labour**					
Labour duration II (min)	44.93±33.99	33.59±27.141	**5.321**	**0.369**	**0.000**
Episiotomy (n, %)					
Yes	78(31.45)	92(12.57)	**46.06**	**0.217**	**0.000**
No	170(68.55)	640(87.43)			
Neonatal birth weight (g)	3358.14±378.0	3287.48±387.227	**2.499**	**0.141**	**0.013**
Weekday delivery (n, %)					
Yes	203(81.85)	605(82.65)	0.081	0.009	0.776
No	45(18.15)	127(17.35)			
Delivery shift (n, %)					
Day shift	126(50.80)	356(48.63)	0.350	0.019	0.554
Night shift	122(49.20)	376(51.37)			

Supplement2 Comparison of postpartum haemorrhage and non-haemorrhage data in training dataset(continued)

**Characteristics**	**Postpartum haemorrhage group (N=248)**	**Non-postpartum haemorrhage group (N=732)**	***t/Z/χ^2***	***Cohen'd s/r/φ***	***P***
**Intrapartum factors of third stage of labour**					
Labour duration III (min)	6.16±4.418	6.03±3.415	0.483	0.033	0.629
Mode of placental delivery (n, %)					
Natural delivery	218(87.90)	691(94.40)	**11.631**	**0.109**	**0.001**
Artificial detachment of placenta	30(12.10)	41(5.60)			
Placenta remnant (n, %)					
Yes	39(15.73)	59(8.06)	**12.09**	**0.111**	**0.001**
No	209(84.27)	673(91.94)			
Delayed umbilical rupture (n, %)					
Yes	117(47.18)	532(72.68)	**53.85**	**0.234**	**0.000**
No	131(52.82)	200(27.32)			
Mother-to-child skin contact (n, %)					
Yes	140(56.45)	463(63.25)	3.618	0.061	0.057
No	108(44.55)	269(36.75)			
**Intrapartum factors of other factors**					
Companionship during labour and childbirth (n, %)					
Yes	156(62.90)	629(85.93)	**61.61**	**0.251**	**0.000**
No	92(39.10)	103(14.07)			
Maternal mobility and position (n, %)			**5.189**	**0.073**	**0.023**
Yes	143(57.66)	481(65.71)			
No	105(42.34)	251(34.29)			

Supplement 3 Comparison of training and test dataset for these 13 variables

Characteristics	Training dataset (N=980)	Test dataset (N=245)	$t/Z/\chi^2$	*P*
**Demographic factors of pregnant women**				
Blood type (n, %)				
Type A+	258(26.33)	55(22.45)	3.545	0.315
Type B+	215(21.94)	47(19.18)		
Type AB+	68(6.94)	18(7.35)		
Type O+	439(44.80)	125(51.02)		
**Demographic factors of midwife**				
Midwife's years of service (years)	11.16±6.55	10.80±6.33	0.851	0.427
**Antenatal factors of present medical history**				
Gravidity (n, %)				
1 time	328(33.47)	91(37.14)	1.175	0.278
⩾2 times	652(66.53)	154(62.86)		
Parity (n, %)				
Primipara	524(53.47)	128(52.24)	0.118	0.731
Multipara	456(46.53)	117(47.76)		
Diagnosis of liver impairment (n, %)				
Yes	8(0.82)	5(2.04)	2.799	0.094
No	972(99.18)	240(97.96)		
Prenatal diagnosis of macrosomia				
Yes	17(1.73)	8(3.27)	2.297	0.130
No	963(98.27)	237(96.73)		
**Antenatal factors of psychosocial factors**				
Degree of childbirth fear (n, %)				
Normal	383(39.08)	103(42.04)	0.937	0.817
Mild	452(46.12)	106(43.27)		
Moderate	121(12.35)	31(12.65)		
Severe	24(2.45)	5(2.04)		
**Intrapartum factors of first stage of labour**				
Antepartum fever (n, %)				
Yes	141(14.39)	41(16.73)	0.853	0.356
No	839(85.61)	204(83.27)		

Supplement 3 Comparison of training and test dataset for these 13 variables((continued)

Characteristics	Training dataset (N=980)	Test dataset (N=245)	$t/Z/\chi^2$	*P*
**Intrapartum factors of second stage of labour**				
Labour duration II (min)	36.56±29.73	35.34±27.42	1.373	0.558
Episiotomy (n, %)				
Yes	171(17.45)	36(14.69)	1.059	0.303
No	809(82.55)	209(85.31)		
**Intrapartum factors of third stage of labour**				
Mode of placental delivery (n, %)				
Natural delivery	908(92.65)	226(92.24)	0.047	0.828
Artificial detachment of placenta	72(7.35)	19(7.76)		
Delayed umbilical rupture (n, %)				
Yes	649(66.22)	160(65.31)	0.074	0.786
No	331(33.78)	85(34.69)		
**Postnatal factors of other factors**				
Companionship during labour and childbirth (n, %)				
Yes	785(80.10)	198(80.82)	0.063	0.802
No	195(19.90)	47(19.18)		

Supplement 4：The specific hyperparameters for each model

To ensure a fair comparison among different algorithms, this paper trained and evaluated five categories of machine learning models on the same dataset(training dataset): Random Forest (RF), Support Vector Machine (SVM), Extreme Gradient Boosting (XGBoost), Artificial Neural Network (ANN), and Logistic Regression (LR). The hyperparameters of all models were set within a reasonable range to ensure the comparability of model complexity and training conditions. The specific configurations are as follows:

```
model =XGBoost Classifier
    n_estimators=400,
    max_depth=4,
    learning_rate=0.05,
    subsample=0.8,
    colsample_bytree=0.8,
    random_state=42,
    n_jobs=-1

model = RF Classifier
    n_estimators=400,
    max_depth=4,
    max_features=0.8,
    bootstrap=True,
     random_state=42,
     max_samples=0.8,
    n_jobs=-1
```

model = SVM Classifier

kernel="rbf",

C=1.0,

gamma="scale",

probability=True,

random_state=42

model = ANN classifier

hidden_layer_sizes=(64, 32),

activation="relu",

solver="adam",

learning_rate_init=0.001,

max_iter=2000,

random_state=42

model = LR classifier

penalty="l2",

C=1.0,

solver="lbfgs",

max_iter=2000,

random_state=42

All models were set with random_state=42 to ensure the reproducibility of the results.

Supplement 5 The results of five-fold cross-validation with five machine-learning algorithms

Model	AUC	Accuracy	Precision	F1 score	Recall
RF	0.828(0.790～0.866)	0.816(0.801～0.832)	0.760(0.700～0.820)	0.549(0.500～0.597)	0.435(0.385～0.485)
SVM	0.814(0.787～0.841)	0.811(0.794～0.829)	0.748(0.679～0.818)	0.537(0.492～0.583)	0.423(0.381～0.464)
XGBoost	0.834(0.801～0.867)	0.813(0.795～0.831)	0.764(0.688～0.839)	0.542(0.494～0.590)	0.431(0.377～0.484)
ANN	0.815(0.783～0.847)	0.808(0.793～0.824)	0.712(0.655～0.769)	0.548(0.498～0.597)	0.454(0.398～0.511)
LR	0.817(0.789～0.844)	0.807(0.785～0.829)	0.710(0.645～0.775)	0.544(0.487～0.600)	0.447(0.390～0.504)

Notes：RF：Random Forest，SVM：Support Vector Machine， XGBoost：eXtreme Gradient Boosting，ANN：Artificial Neural Network，LR：Logistic Regression

Supplement 6 Interaction analysis and introduction

Supplement 6-a Interaction analysis of the midwife's years of service and childbirth fear

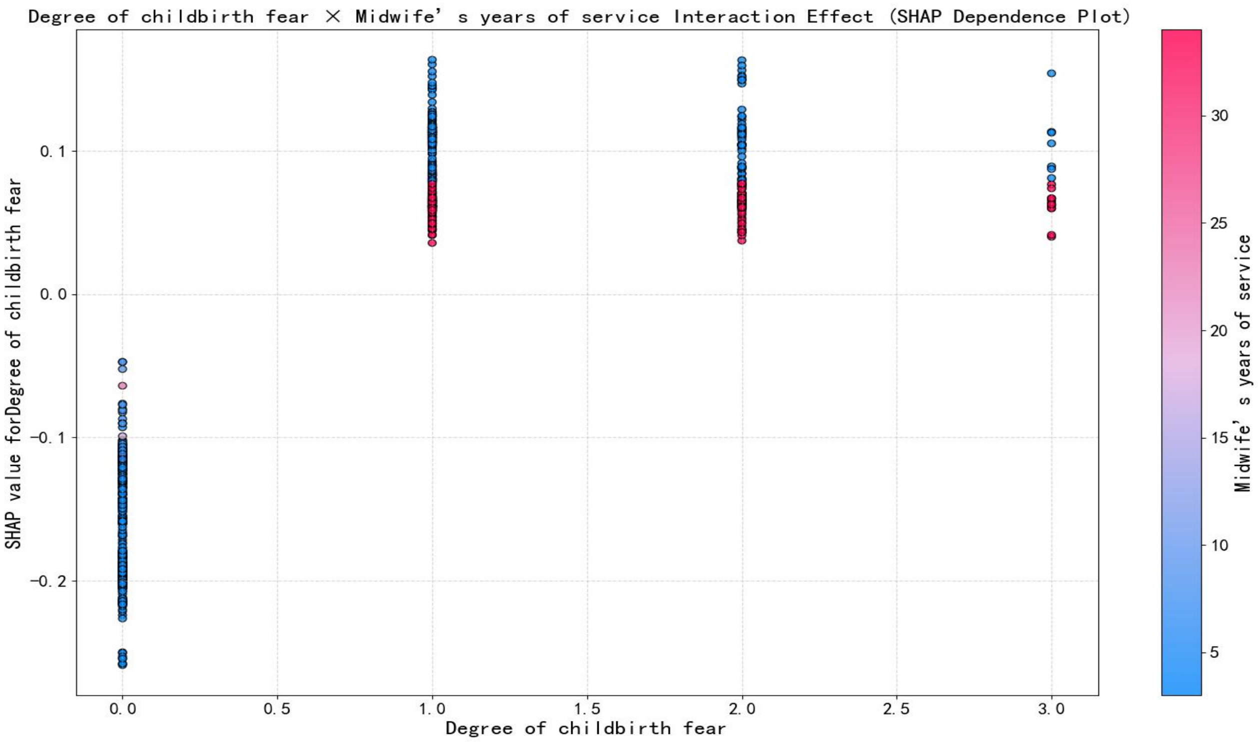

Fig 6-a SHAP dependence plots

Note:When the fear of childbirth score is 0 (indicating no fear), the SHAP values are concentrated predominantly in the negative range, suggesting that the absence of such fear may reduce the risk of postpartum hemorrhage.When the fear-of-childbirth score ranges from 1 to 3 (indicating mild, moderate, or severe fear), the SHAP values are chiefly positive, suggesting that fear of childbirth may increase the risk of postpartum hemorrhage.The midwives' years of experience moderate the effect of fear of childbirth. When there is no fear, points tending toward red (Midwife's years of service ＞ 10years) and those toward blue (Midwife's years of service ≤ 10years) overlap considerably along the vertical axis, indicating that experience does not markedly change how the absence of fear influences the predicted outcome.When fear is present, red points lie lower than blue points, indicating that greater experience attenuates the extent to which fear of childbirth raises the predicted risk of postpartum hemorrhage.

Table 6-a Interaction analysis of the midwife's years of service and childbirth fear using LR model

Childbirth fear	Midwife's years of service	Events / Total	Crude OR [95% CI]	Adjust OR[a] [95% CI]
No	≤10years	50/159	1 [Reference]	1 [Reference]
No	＞10years	67/241	0.88 [0.58, 1.34]	0.87 [0.76, 1.10]
Yes	≤10years	64/82	2.48 [1.57, 3.92]	2.78 [1.82, 4.23]
Yes	＞10years[b]	129/433	0.95 [0.65, 1.38]	0.98 [0.71, 1.71]
Multiplicative Interaction			OR=0.43[95%CI: 0.21, 0.89]	
Additive Interaction			RERI=−1.42[95%CI: −1.96, 0.18]	
			AP=−1.50[95%CI: −3.06, 0.07]	
			S=−0.04[95%CI: 0.3,0.22]	

Note: a:adjusted variables included gravidity, parity, episiotomy, mode of placental delivery, diagnosis of liver impairment, prenatal diagnosis of macrosomia, antepartum fever, blood type, and delayed cord clamping. b: reference group in the addilive interaction. OR:odds ratio, Cl:confidence interval, RERl:relative excess risk due to interaction, AP:attributable proportion,S:synergy.

Supplement 6-b Interaction analysis of companionship during labour and childbirth fear

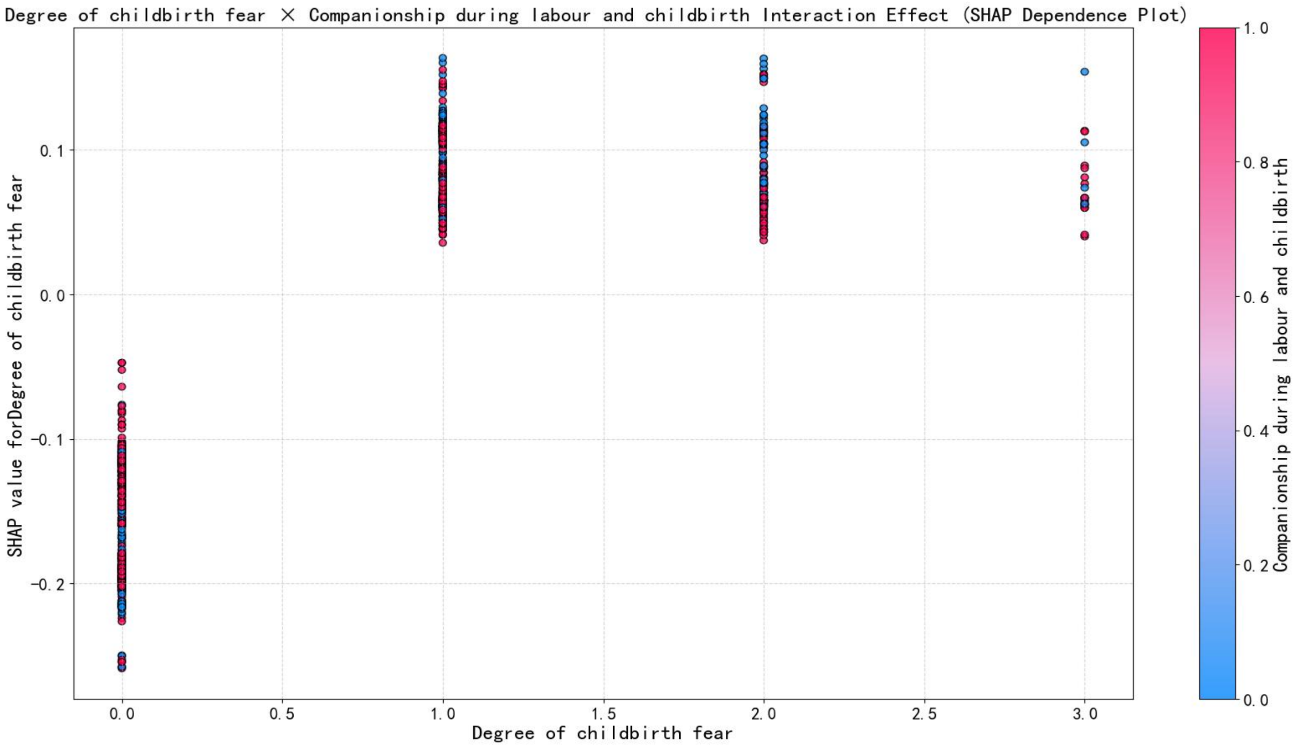

Fig 6-b SHAP dependence plots

Note:for a detailed description of the fear of childbirth, see Figure 6-a.Companionship during labour moderates the effect of childbirth related fear on the predicted risk of postpartum haemorrhage. In the absence of fear, the SHAP values for parturients with (red points) and without (blue points) continuous companionship overlap almost completely along the vertical axis, indicating that companionship does not materially alter the influence of a zero fear score on model predictions. When fear is present, the red points are systematically lower than the blue points, demonstrating that continuous companionship attenuates the positive SHAP values associated with fear, and thus mitigates the increase in predicted postpartum haemorrhage risk attributable to childbirth fear.

Table 6-b Interaction analysis of companionship during labour and childbirth fear using LR model

Childbirth fear	Companionship	Events / Total	Crude OR [95% CI]	Adjust OR[a] [95% CI]
No	No	37/103	1 [Reference]	1 [Reference]
No	Yes	144/ 409	0.98 [0.64, 1.69]	0.96 [0.64, 1.56]
Yes	No	55/86	1.78 [1.26, 2.44]	1.83 [1.4, 2.21]
Yes	Yes[b]	74/317	0.65 [0.59, 0.89]	0.75 [0.69, 0.97]
Multiplicative Interaction			OR=0.427[0.20,0.71]	
Additive Interaction			RERI=−1.11[−1.89,−0.33]	
			AP=−1.71[−2.96,−0.45]	
			S=-0.46[-0.96,0.04]	

Note: a:adjusted variables included gravidity, parity, episiotomy, mode of placental delivery, diagnosis of liver impairment, prenatal diagnosis of macrosomia, antepartum fever, blood type, and delayed cord clamping. b: reference group in the addilive interaction. OR:odds ratio, Cl:confidence interval, RERl:relative excess risk due to interaction, AP:attributable proportion,S:synergy.

Supplement 6-c Interaction analysis of companionship during labour and the duration of the second stage of labour

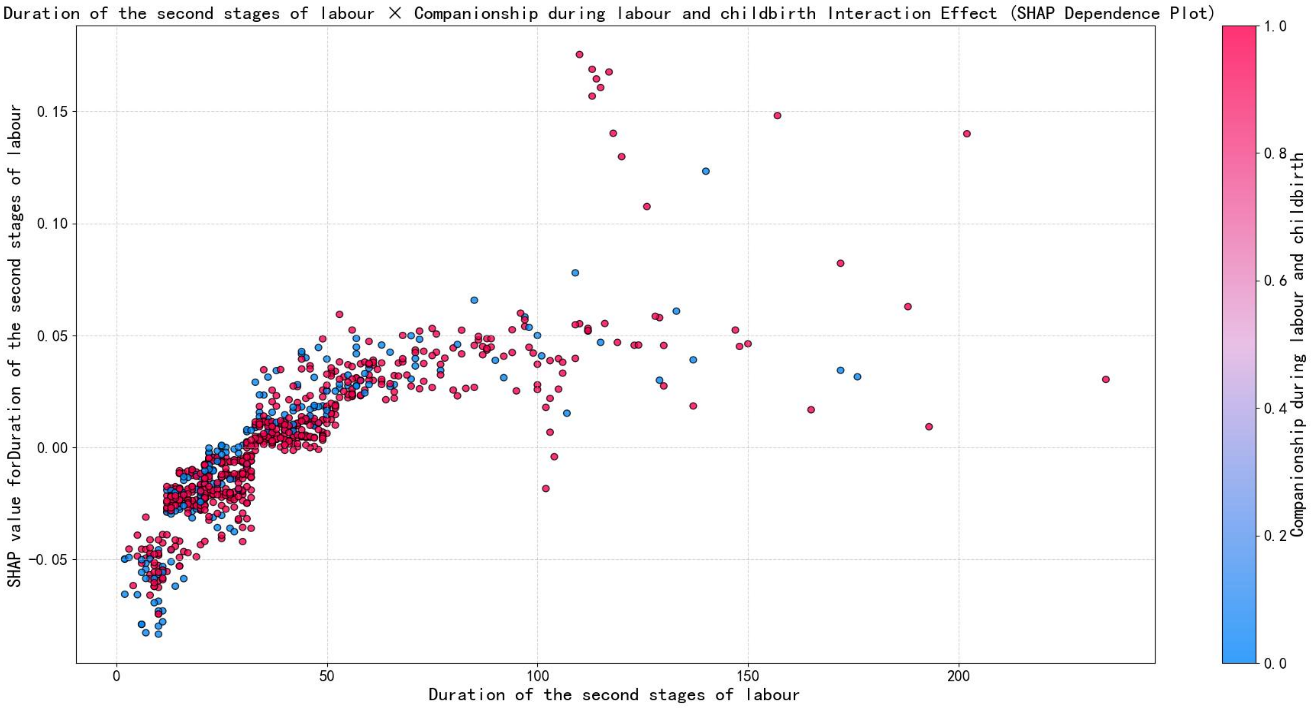

Fig 6-c SHAP dependence plots

Note:When the duration of the second stage of labour is less than 30 minutes, the SHAP values are mostly negative, suggesting that a duration of less than 30 minutes reduces the risk of postpartum haemorrhage. When the duration is greater than 30 minutes, the SHAP values become significantly positive, indicating an increased risk of postpartum haemorrhage, which tends to stabilise when the duration exceeds 70 minutes. Labour companionship has a moderating effect on the duration of the second stage of labour. When labour lasts ≤30 minutes, the SHAP values for cases attended by have companionship (red points) and those attended by no companionship (blue points) are almost perfectly aligned along the vertical axis, indicating that companionship does not meaningfully alter the directional impact of short labour on the predicted outcome. Conversely, when labour extends beyond 30 minutes, the red points lie systematically below the blue points, demonstrating that have companionship tenure attenuates the positive SHAP values associated with prolonged labour and thus mitigates the concomitant rise in predicted risk.

Table 6-c Interaction analysis of companionship during labour and the duration Duration Of the second stage of labour using LR model

Duration Of the second stage of labour	Companionship	Events / Total	Crude OR [95% CI]	Adjust OR[a] [95% CI]
≤30minutes	No	74/155	1 [Reference]	1 [Reference]
≤30minutes	Yes	142/480	0.62 [0.48, 1.02]	0.72 [0.57, 1.12]
＞30minutes	No	55/90	1.28 [0.83, 1.68]	1.18 [0.95, 1.38]
＞30minutes	Yes[b]	39/190	0.43 [0.28, 0.67]	0.63 [0.45, 0.83]
Multiplicative Interaction			OR=0.54[0.30, 0.99]	
Additive Interaction			RERI=−0.47[−0.82, 0.12]	
			AP=−1.09[−1.60, 0.31]	
			S=1.70[0.87, 3.87]	

Note: a:adjusted variables included gravidity, parity, episiotomy, mode of placental delivery, diagnosis of liver impairment, prenatal diagnosis of macrosomia, antepartum fever, blood type, and delayed cord clamping. b: reference group in the addilive interaction. OR:odds ratio, Cl:confidence interval, RERl:relative excess risk due to interaction, AP:attributable proportion,S:synergy.

Supplement 7 The existing postpartum haemorrhage prediction models for vaginal delivery

Author, year, (Country)	Diagnosis of PPH	Models	Collect feature variables	Variables in model	Performance
Getachew Assefa，2023 （Japan）	Vaginal delivery>1000ml within 24 h after birth	LR	clinical variables	pregnant gestation of labor, the maternal weight upon admission of labor, and the maternal weight before pregnancy	AUC 0.708, accuracy 0.686 false positive rate 0.312 False negative rate 0.398
Jia Liu，2022 （China）	Vaginal delivery>500 ml within 24 h after birth	LGB + LR (Optimal models)	prenatal and intrapartum attributes,biochemical test, maternal vital signs within 2h after delivery, uterine contraction curve	hematocrit (%), shock index, frequency of contractions(min-1), white blood cell count, gestational hypertension, neonatal weight(kg), time of second labor(min), mean area of contractions (mmHg), total amniotic fluid (mL), and body mass index (BMI).	AUC 0.803 Brier 0.061 F-measure 0.845 SE 0.694 SP 0.800
Line Bihan，2022 （ France）	Vaginal delivery>500 ml within 24 h after birth	LR	clinical and biological data	pre-eclampsia, antepartum bleeding, multiple pregnancy, labor duration ≥ 8h, macrosomia, episiotomy, platelet count < 150 Giga/L and aPTT ratio≥1.1	AUC0.66

Supplement 7 The existing postpartum haemorrhage prediction models for vaginal delivery（(continued)

Author, year, (Country)	Diagnosis of PPH	Models	Collect feature variables	Variables in model	Performance
Chenning Liu，2021（China）	Vaginal delivery>1000 ml within 24 h after birth	LR	demographic,pre-gestational, medical, and obstetrical factors	previous cesarean section, history of PPH, invitro fertilization, anemia, intrauterine death, prolonged labor,low-lying placenta, placental abruption, placenta accreta spectrum, and macrosomia	C-index 0.86
Chunxue Yang，2023（China）	Vaginal delivery>500 ml within 24 h after birth	LR	demographic and clinical information,prelabor blood indicators	pregnancy-induced hypertension, a lower prelabor FIB, a longer third stage of labor, and a higher birth weight for the newborn	AUC 0.873

Supplement 8 Description of the variables

Variables	Definition	Units or categories
Midwife's years of service	The total number of years from the date on which the midwife obtained the qualification in "Single Spontaneous Vaginal Delivery" to the study start date.	Year
Degree of childbirth fear	A continuous measure reflecting the intensity of fear specifically related to the upcoming birth assessed by CAQ(see Section 2.4.1 for details）	Normal，Mild，Moderate，Severe
Duration of the second stages of labour	The interval in minutes from the first documentation of complete cervical dilatation (10 cm) to the complete delivery of the infant	Minute
Companionship during labour and childbirth	Continuous one-to-one support provided by a designated family member and the midwife from cervical dilation of 3 cm until completion of delivery	Yes or No
Blood type	The maternal ABO group (A^+, B^+, AB^+, or O^+) determined by routine serologic testing at the last antenatal visit	Type A^+、B^+、AB^+、O^+
Delayed cord clamping	Cord clamping is deferred for at least 1–3 minutes or until pulsation in the umbilical artery has ceased before the cord is ligated.	Yes or No
Diagnosis of liver impairment	At any point during pregnancy, serum alanine aminotransferase (ALT) or aspartate aminotransferase (AST) was ⩾ 2×the upper limit of normal, or total bilirubin was ⩾ 1.5× the upper limit of normal, after exclusion of other established causes	Yes or No
